# Geoacumulation of Heavy Metals in Sediment of the Fluvial–Lagoon–Deltaic System of the Palizada River, Campeche, Mexico

**DOI:** 10.3390/ijerph17030969

**Published:** 2020-02-04

**Authors:** Gabycarmen Navarrete-Rodríguez, María del Refugio Castañeda-Chávez, Fabiola Lango-Reynoso

**Affiliations:** Tecnológico Nacional de México/Instituto Tecnológico de Boca del Río, Laboratorio de Investigación en Recursos Acuáticos LIRA, Kilómetro 12, Carretera Veracruz-Córdoba, Boca del Río 94290, Mexico

**Keywords:** pollution sources, sediments, Palizada River, heavy metals

## Abstract

The fluvial–lagoon–deltaic system of the Palizada River in Campeche is an ecosystem of socioeconomic and ecological importance. It is justifiable to carry out studies in this system due to its connection with another larger ecosystem called the Términos Lagoon. The objective of this investigation was to analyze the concentration of Pb and Cd in sediments of the fluvial–lagoon–deltaic system of the Palizada River and to determine, with this, the contamination index of these metals. Cd showed the highest concentration in sampling sites and climatic seasons with respect to Pb, with a maximum value of 53.9 ± 5.0, while the Pb concentration was 10.4 ± 0.2 μg∙g^−1^. The same tendency was present with pollution and geoaccumulation indexes; here, the Cd index stands out. The enrichment of heavy metals was identified through the accumulation of Cd and Pb; such a process was evaluated through the geoacumulation index (Igeo). The results of this indicated that the contamination of these elements is mainly of anthropogenic origin. This element represents an ecological toxic risk due to the chronic presence of heavy metals in a priority area for the conservation of aquatic and terrestrial biota such as the Palizada system, owing to its high toxicity even at low concentrations. Thus, it is important to evaluate its sublethal effects in the organisms that inhabit this system, which requires the implementation of integral monitoring.

## 1. Introduction

Sediments are important deposits of pollutants such as heavy metals and pesticides. They play an important role in the remobilization of pollutants in aquatic systems under favorable conditions and in water–sediment interactions [1]. In the case of heavy metals, these elements reach rivers when their partial dissolution occurs, their transfer to water and living organisms is the result of physical and chemical changes in river conditions [2]. In water, most suspended particles tend to bind to heavy metals and form complexes, which precipitate in the sediment where they accumulate gradually [3].

The quality of coastal lagoon sediments depends on anthropogenic activities related to land use and proximity to agricultural areas [4]. However, the presence of these in the environment occurs naturally, since all marine soils and sediments contain trace metals as natural constituents, and these are leached into water at low concentrations [5]. In aquatic ecosystems, heavy metals and trace are incorporated from natural and anthropogenic sources. These are considered some of the most important environmental pollutants to be known due to their toxicity, persistence, and tendency to accumulate in aquatic organisms [6].

The dynamics of heavy metals movement between environmental matrices sediment and water becomes a cycle of absorption and release, since once trace elements are released in the water column, they bioaccumulate in aquatic sediments in greater proportion. The dynamics of these elements depend on environmental conditions such as the seasonal variations, pH, microorganisms, sediment reduction, and oxidation potential; the presence of metals in water represents a potential risk for aquatic organisms that are in direct contact with the sediment [7,8,9]. Metals such as Pb and Cd not having essential biological functions are considered relatively rare in the environment. They have a source of anthropogenic origin and are metals of greatest risk to human health and biota because they are highly toxic in low concentrations [2,10]. Meanwhile, other metals, for example Cu, although toxic in high concentrations are at low concentrations an essential element for all organisms, including animals and humans [11]. Elements such as Pb and Cd are indicators of environmental quality in coastal systems. The dynamics of mobilization of these pollutants in coastal areas is important, since the increase in concentrations may be due to anthropogenic causes carried out in the region due to the contribution of activities such as agriculture and livestock that are carried out on the banks of the Palizada River and Laguna de Terminos. In the case of Cu, these are due to agricultural fungicides, while industrial activities are the main source of the Pb and Cd cases [2].

In estuaries and coastal wetlands, sediments protect a variety of pollutants, which are mostly heavy metals. Therefore, they can serve as an enriched source of these elements for benthic organisms [10]. Organisms exposed to heavy metals can be induced to undergo physiological and biochemical changes [11]. The process of mobilization of heavy metals in sediment will depend mainly on the composition of the sediment, including its content of organic matter, size, and grain texture [12,13,14]. The importance of analyzing the concentration of heavy metals in sediments of fluvial–lagoon systems in Mexico is because the results of this work will indicate their quality, as well as the long-term effect on biota and public health. This work aimed to establish the concentration of metals in sediments of the fluvial–lagoon–deltaic system of the Palizada River in Campeche and thus to determine its quality according to the index of contamination of the elements evaluated.

## 2. Materials and Methods 

### 2.1. Study Area

The fluvial–lagoon–deltaic system of the Palizada River is located in the southwest portion of the hydrological basin of the Términos Lagoon, between the geographic coordinates 18°19′13″ and 18°29′04″ north latitude and 91°44′36″ and 91°51′31″ west longitude [2,15,16]. The Palizada River is a tributary of the Usumacinta River; it is separated from the latter at the point known as Boca de Amatitlán, which is located approximately 25 km from the Palizada city. The river of the same name traverses southwest–northeast through the municipality, and its flow is unloaded in the Boca Chica of the Términos Lagoon [17,18]. Términos Lagoon being a coastal type, forms part of this lagoon fluvial system. It is one of the most widely studied ecosystems, including the rivers that converge in it. It is worth noting that it was decreed as a protected natural area in 1994 [2]. In addition, it is important to mention that the Palizada River system contributes an important flow volume to the Términos Lagoon with 240 m^3^∙s^−1^, followed by the Chumpan River with 35 m^3^∙s^−1^ and the Candelaria River with 2 m^3^∙s^−1^ [15]. There are three prevailing climatic conditions a year in the region of the system. The turbulence season is from October to January, which is when there are strong north and northwest winds with an average speed of 8.3 m^3^∙s^−1^ [15,18].

**Figure 1:**
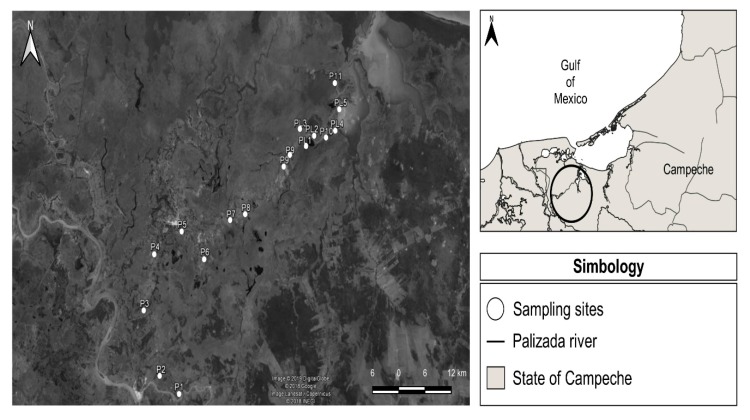
Sampling sites to sediment of the fluvial–lagoon–deltaic system of the Palizada River, Campeche, Mexico.

In the dry season from February to May, winds blow predominantly from the northwest at speeds of 4–6 m∙s^−1^ and the currents in the lagoon are overcome by the tide, which produces a greater seawater input. Whereas, in the rainy season from June to September, there is an increase in water supplied by the rivers [19,20]. Regarding sampling sites for sediment of the fluvial–lagoon–deltaic system of the Palizada River in Campeche, a total of 18 sampling sites were selected. During this study, a total of 162 samples were analyzed in triplicate and collected three times during the year (Figure 1). Samples were collected with a dredge and stored in polyethylene bags to be transported to the laboratory at temperatures of 4 °C; then, these were preserved in freezing.

### 2.2. Laboratory Analysis

Samples obtained in the field were processed in the Instituto Tecnológico de Boca del Río (ITBOCA), in areas of the Aquatic Resources Research Laboratory. Once the samples were extracted, they were stored in hermetically sealed food-grade polyethylene bags and kept frozen until freeze-dried in a Thermo Savant ModulyOD-114 unit for 72 h at −49 °C and a vacuum pressure of 36x10-3 mbar. The lyophilized samples were stored in a desiccator until acid digestion analysis.

### 2.3. Acid Digestion in Microwave

The preparation of the material used in the analysis was carried out in accordance with the specifications for heavy metal analysis NOM-117-SSA1-1994 [21]. The laboratory material was washed using neutral phosphate-free Extran^®^ soap; then, it was soaked in a solution of nitric acid HNO_3_ (JTBaker^®^) at 20% for 24 h, after which it was washed with deionized water, dried with hot air, and finally stored. The digestion of each lyophilized sample was carried out in microwave equipment CEM Mars 5 (CEM, Corporation Mathews, NC, USA). Using 0.5 g of sample, in addition 9 mL of reactive grade HNO_3_ grado reactive (JTBaker^®^) was added. Then, two ramps with a pressure of 120 and 100 PSI were programmed in a microwave at a temperature of 150 °C and 190 °C for 5 and 10 min, respectively. 

The digestion was performed by batch process with a blank sample with deionized water, and a positive control sample selected in the one random batch was analyzed to confirm certain processing samples. Once the microwave digestion was complete, Millipore^®^ nitrocellulose filters of 0.45 μm were used, and the filtrate was made up to a volume of 25 mL with deionized water (Milli-Q); the final extract was deposited in amber polyethylene vial and stored at a temperature of 4 °C. To perform the quantification of heavy metals, a Thermo Scientific iCE 3500 AAS (Thermo Scientific^®^, China) was used. Certified standards, High Purity Standards^®^ (High-Purity Standards, Charleston, SC, USA), with a concentration of 1000 µg∙mL^−1^ in 2% DE HNO_3_ were used in the preparation of the calibration curve for the quantification of metals. The wavelength used for the wavelength reading of Pb was 217 nm, while that used for the wavelength readings of Cd and Cu was 324.8 nm. In addition, a correlation coefficient greater than 0.96 was obtained, for which standards elaborated at known concentrations were used, with an adjusted range of a lower to higher concentration close to the analyte. The precision and accuracy of the results were evaluated from the determining recovery repeatability of 10 samples of known concentration for each metal.

### 2.4. Heavy Metal Enrichment: Pollution Load Index (PLI) for Heavy Metals and Geoaccumulation Index (Igeo)

For the calculation of the Contamination Factor metal (*CF*), the relationship between metal content in a sediment sample and normal concentration levels was considered; based on this, the level of contamination of the sediment was determined. To calculate the *CF* of sediments in the study area, the contamination of trace metals was taken into account from the global average crustal, according to background values. It is considered that this factor will reflect the enrichment of the metal in sediment when *CF* > 1 for a particular metal, meaning that the sediment is contaminated by the element. If *CF* < 1, it expresses that there is no or low contamination of the metal, making contamination a product of natural and anthropogenic inputs. The range of 1 ≤ *CF* ≤ 3 was used for moderate contamination, 3 ≤ *CF* ≤ 6 was used for a considerable contamination, and *CF* > 6 was used for a very high contamination [22,23,24]. The above was made according to the following relationship:

(1) CF=Contamination factor metal content in sedimentBase metal value

The environmental quality of the sediments was evaluated with the Pollution Load Index (*PLI*) integrated for heavy metals, which is also known as the Metal Content Index with the acronym (*MCI*). Here, n is the number of metals analyzed, and *CF* is defined as the concentration of a metal in contaminated sediment divided by the normal value in an uncontaminated environment (background values) [25,26].

(2) $$PLI={\left(CF1\times CF2\times CF3\ldots CFn\right)}^{\left(\frac{1}{n}\right)}$$

The enrichment of sediments with metallic elements was analyzed by means of the geoaccumulation index (*Igeo*); this is a quantitative measure of contamination of metals in aquatic sediments, comparing the contents of current metals with preindustrial levels, and it was introduced by Müller [27,28]. This indicator reflects the degree of sediment contamination by a metal [29]. The Igeo provides a simple and fast method to determine the extent of sediment contamination of a lake or riverbed by loading trace elements into sediments above background values, but it does not provide further information regarding the mobilization and bioavailability of the trace element [28,30]. The value of the geoaccumulation index is described by the following equation:

(3) $$Igeo=log_{2\;\;}\frac{\left[Cn\right]}{k\;\times Bn}$$

where Cn is the measured concentration of the element n (number of metals) in the soil tested and Bn is the geochemical background value of the element n in average crust. Here, the average metal content in the Earth’s crust of (X) is measured in mg∙kg^−1^, while *k* = 1.5, to consider possible variations in the background data due to the lithogenic effects (constant factor) and take into account natural fluctuations of a substance given in the environment, as well as very small anthropogenic influences [30,31]. Bn reference values were taken from Huheey [32]. Seven grades or classes of the geoaccumulation index were proposed with the following types: Class 0 (practically uncontaminated): *Igeo* < 0; Class 1 (uncontaminated to moderately contaminated): 0 < *Igeo* < 1; Class 2 (moderately contaminated): 0 < *Igeo* < 2; Class 3 (moderate to highly contaminated): 2 < *Igeo* < 3; Class 4 (highly contaminated): 3 < *Igeo* < 4; Class 5 (highly contaminated to extremely contaminated): 4 < *Igeo* < 5; Class 6 (extremely contaminated): 5 < *Igeo*. Finally, Class 6 is an open class and comprises all values of the index higher than Class 5 [29,33]. 

The concentrations of heavy metals in sediment were studied by sampling site and season; these were analyzed with Statistica 7.0 software (StatSoft, Inc. Tulsa, OK, USA), and a one-way analysis of variance was also used. A multiple comparison of means using the Tukey test was performed afterwards to determine at the level of significance α = 0.05 for the significant difference statistics (*p* < 0.05) the significant difference between concentration of heavy metals for site sampling, season, and *Igeo*.

## 3. Results and Discusion

### 3.1. Sources of Heavy Metals in the Palizada River System

The sediments of an aquatic system reflect the environmental quality of an ecosystem; they are fundamental in the dynamics of distribution and accumulation of pollutants such as heavy metals [2]. These elements are released to the environment and have contact with aquatic systems through a process of wet or dry deposition, erosion, and/or by the direct downloads of anthropogenic activities, which are carried out in collective areas [34]. In the Palizada system, in addition to traditional fishing, there is also industrial activity as it is the extraction of crude oil, which is certainly the main source of economic income in the region [35]. In contrast, it is highlighted that on both sides of the Palizada, Chumpán, and Candelaria rivers in Campeche, the main economic activity is agriculture, which as a consequence of the use of agrochemicals provides heavy metals to the streams of these rivers [36].

An important factor in the distribution of heavy metals in the Palizada River corresponds to the hydrological conformation of the fluvial–lagoon–deltaic system of the river, since it is made up of numerous internal lagoons, which are influenced by the anthropogenic contribution of livestock and agricultural activities that take place south of its margins [6,37]. The analysis of heavy metals in a portion of the surface basins represents an indicator of the behavior of these contaminates; they are a baseline of research for the development of control and mitigation strategies. Research on heavy metals in sediments in the river basins of Mexico has focused very punctually on certain rivers but without continuous monitoring of the region in question, such as for example the work done in the Grijalva basins [38], the Puebla River that includes the Alseseca and Atoyac rivers and the Valsequillo dam [39], the Atoyac River [40,41], and the Tecate River [42]. However, the distances between regions and periods coincide with the need for continuous monitoring in a Palizada river lagoon system [2,6], and research work has been carried out discontinuously in the study area [36]. The importance of evaluating the current concentration of metals that represent a public health risk such as Pb and Cd should be highlighted.

Different types of heavy metals inputs to the aquatic ecosystems show their wide distribution in the system. This is the case of the fluvial–lagoon–deltaic system of the Palizada River, where the Pb and Cd were identified in all the sampling sites analyzed in the present investigation. The contribution of metals is also associated with the dynamics of the Palizada river system. As a consequence, the suspended ground materials generate an increase in the turbidity and contribute with a great amount of sediments to the Términos lagoon [35]. In accordance to the above mentioned, the Palizada River contributes with an average percentage of 92% of the total contribution of heavy metals that are downloaded in the lagoon and, as such, variations in the proportion of each metal concentration are presented [35,43].

The sediments can accumulate heavy metals that reach aquatic environments, in which changes in physicochemical conditions can be remobilized; in doing so, releasing these elements in the water column, these contributions can be transferred through the food chain [2,36]. The Palizada system interacts with surrounding aquatic bodies, presenting dynamics of material distribution to the Términos Lagoon, in which the water column, in its totality, acts as a sink for nutrients due to a high production of particles, dissolved organic matter, and a reduced export of material to the Gulf of Mexico [44]. As a consequence, in the Términos lagoon, a spatial and temporary heterogeneity of water–sediment flows predominates, with peaks in the mineralization rates during the rainy season, relating significantly to the content of organic matters in sediments [45]. The above demonstrates the relationship between the Palizada River and the Términos Lagoon, where there is a correlation in the content of organic matter, the texture of the sediment, and the content of heavy metals [36]. This is a result of the proportions between the following: clay–organic matters (OM); sand–vanadio; OM–Cu, and Cu–clay. Therefore, it is observed that the mobilization and accumulation of heavy metals is influenced by the relationship between the Palizada River fluvial lagoon system, the Términos Lagoon, and the Gulf of Mexico, indicating an interrelationship between the continental and coastal systems in the transportation of pollutants. 

#### 3.1.1. Lead Concentration (Pb)

**Figure 2:**
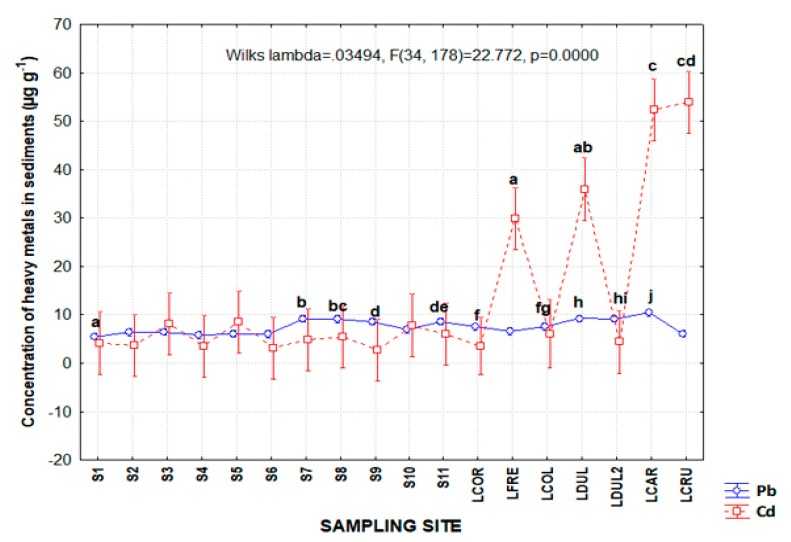
Concentration of heavy metals in sediment (µg∙g^−1^) from sampling sites of the fluvial–lagoon–deltaic system of the Palizada River in Campeche. The different literals indicate significant differences between sampling sites. Abbreviations: LCOR-Corcho lagoon; LDUL-Dulce lagoon; LCAR-Carmen lagoon; LLAR-Larga lagoon; LCOL-Colorada lagoon; LCRU-Cruces lagoon; LFRE-Frente lagoon. Different literals such as “a to j” indicate significant differences between sampling sites for each metal.

Pb presence and concentration in aquatic ecosystems can be related to different sources. For the Palizada River system, the sources are the high number of industrial activities such as the exploration and extraction of hydrocarbons in the coastal area adjacent to the Términos Lagoon; heavy metals associated with this activity can enter the lagoon [37]. The Palizada, Chumpán, and Candelaria rivers, as well as some inland lakes such as the Pom, Atasta, Corte, San Carlos, Este, Balchacah, and Panlau, which flow to the south of the Términos Lagoon, represent a source of agrochemicals, among other pollutants [6]. In relation to the above, the presence of Pb in the environment, may be associated with its old uses in the area, with types of gasoline, paint, and pesticide, which have had a significant impact on the amount of lead found in the ground [46]. Once Pb is incorporated, it adheres strongly to particles in the soil and remains in the upper layer of the same. Subsequently, when these soil particles are mobilized by rainwater, small amounts of this element can be dragged into aquatic bodies, entering rivers, lakes, and streams [46]. In sampling sites analyzed in the Palizada river system, the maximum Pb concentrations were presented in the following points: LCAR with 10.4 ± 0.2, followed by LDUL2 with 9.1 ± 0.8; LDUL with 9.2 ± 0.6; S7 with 9.1 ± 0.6; and S8 9.1 ± 0.5 μg∙g^−1^ (Figure 2). It has also been identified that the enrichment and pollution processes with heavy metals such as Cd, Pb, Ni, and Cr in the sediment of the Pra River basin in Ghana represented an indicator of the development of anthropogenic activities in the basin [34].

Lead is an element that can remain attached to sediment and water particles for many years; this occurs mainly because the Pb adheres to the soil particles and its molivilization in it depends on the type of Pb compound and the characteristics of the soil, as organic matter and soil texture are related to the concentrations of Pb, Cu, and Cd [2,36,46]. Heavy metals in perennial rivers present in dissolved forms or associated with fine sediments can be transported over long distances, depending on the flow competition [47]. Therefore, the dynamics of Pb at the sites and times of the year are related to factors of the metal, the characteristics of the sediment, and the circulation of water in the river. Regarding permissible values for Pb in sediments for Mexico, there is no reference value, it is not clear that the EPA has determined that Pb is probably carcinogenic in humans, since this can affect almost all organs and systems in the body as well as the nervous system of children and adults [46].

The minimum concentrations were in the following sites: S1 (5.5 ± 1.0); S4 (5.7 ± 0.8); S6 (6.0 ± 1.6); and LCRU (6.0 ± 0.6). The sampling sites showed significant statistical differences (*p <* 0.05) through a comparison of means made with Tukey, with Mean Squared Error (MSE) values of 0.78672 for S1, S7, S9, LCOR, LDUL, and LCAR (Table 1). However, there was an overlap of means with sites S8 and S11, among others. The concentrations reported in this investigation were higher than the values in other studies. In the Muthupet Lagoon in India, average concentrations of Pb presented significant statistical differences (*p <* 0.05) with a range that varied from 0.3 to 1.2 μg∙g^−1^ [48]; the reported variations may be associated with the sources of this metal. In addition, in Mbaa River, the Pb showed the same tendency, with a higher concentration of this element in sediments with respect to water. The authors reported an average concentration of Pb in sediment of 8.23 ± 0.23 mg∙kg^−1^, whereas in the water samples, it was 2.08 ± 0.02 mg∙kg^−1^; this indicates the affinity of Pb to accumulate in sediment and the importance of assessing its behavior in ecosystems such as rivers [49].

Pb concentrations in the rainy and north winds seasons showed significant statistical differences (*p* < 0.05) according to the Tukey test of multiple comparisons and an MSE of 2.4939 was obtained. The maximum concentration of this metal was in the dry season with 8.2 ± 1.3, while the minimum was presented in the north winds season with a concentration of 6.6 ± 1.7 μg∙g^−1^. Variations in metal concentrations per season occur because during the dry season, there is an increase in the rate of evaporation and salinity, which is associated with high temperatures [15]. While in stormy weather (north winds), the winds help to mix the water column and its nutrients and with this, the resuspension of sediments and also with the washing of the soil surface [15,34].

Furthermore, in the case of Fe, an increase in its concentration was identified in the Palizada River system during the dry season, suggesting the existence of evaporation phenomena and the low mobility of the sediments [2]. In addition, during this season, there is a decrease in the flow of water from the rivers to the Términos Lagoon; this favors the entry of seawater [15]. The aforementioned reflects that the Palizada River, during the north winds season, presents a greater interaction of continental and marine water, this allows to register this aquatic body as an extremely dynamic system [50].

In addition, researchers have emphasized the difficulty of differentiating between the origin of anthropogenic contributions and natural contributions of an aquatic ecosystem, highlighting that the increase in concentrations between seasons is an important element for pollution analysis [2]. In the same study area, the importance of the climatic season and sediment characteristics as essential elements for the mobilization and transport of heavy metals was underlined, stressing the influence of the season with variations in concentrations of elements analyzed in the Palizada River [6]. Separately, in the Muthupet Lagoon, Pb concentrations presented a significant statistical difference (*p* < 0.05), as well as a range of 0.3 to 1.2 μg∙g^−1^ [48]; these average concentrations were lower than those obtained in the present investigation. The maximum Pb value was 1.2 μg∙g^−1^ in Station 1 of the monsoon season, and the minimum value of 0.3 μg∙g^−1^ was observed in Station 2 of the post-monsoon season, while an average range of lead was recorded with 3.4 μg∙g^−1^ at the station during the entire study period [48]. The Passur River in Bangladesh showed an average Pb concentration of 6.9, with maximum values of 12.2 in June and 11.7 mg∙kg^−1^ in May. Meanwhile, in the remaining months, the concentrations of this element had the following distribution: 1.0, 1.1, 6.4, and 8.9 mg∙kg^−1^ for January, February, March, and April, respectively [51]. Differences in heavy metal concentrations between seasons can be associated with variations in environmental conditions and the discharges of pollutants. 

The existence of seasonal variation in the levels of metal accumulation in the environmental matrices of water, sediments, and biota associated with the characteristics of each environmental matrix has been reported [52]. The concentrations in water and sediments are generally higher in summer than in autumn, and the discharge of leachates in the summer from streams should be considered. Meanwhile, the differences in metal concentration can be attributed to the spatial and temporal heterogeneity of heavy metal distribution (Table 2), especially when related to the variability introduced by wastewater and fluvial discharges [53]. 

Researchers confirmed that the climatic season has a high influence on the variability of metal concentrations in their analysis [6]. Researchers also reported that the highest levels of Cu, Fe, and Mn were found during the dry season, which may be due to the occurrence of significant evaporation phenomena in the area. In the Gorgan Bay in Iran, researchers also noticed that the concentrations of metals such as Al, Cu, Fe, Ni, Pb, and Zn presented significant statistical differences (*p* < 0.05) due to the seasonal and spatial variations in sites of sampling [54]. Meanwhile, the deposition of heavy metals takes place after a period of time and at a greater distance from the discharge zone, which suggests that there is mobilization and balance between the two matrixes [55]. Pb concentrations between climatic seasons in this investigation showed an opposite behavior when registering the maximum concentration in the dry season (Table 2). 

#### 3.1.2. Cadmium Concentration (Cd)

The presence of Cd in the ecosystems is explained by several sources, emphasizing that some fertilizers contain it and it is filtered to the soil during its application in crops [2,56]. The different sources of Cd origin and the characteristics of the sediment are associated with the maximum concentration values that appear in sampling sites. Cd presented a maximum of 53.9 ± 5.0 μg∙g^−1^ at the Cruces Lagoon site (LCRU), while the Pb maximum value was 10.4 ± 0.2 μg∙g^−1^ at the Carmen Lagoon site (LCAR). According to the above, Cd adheres strongly to organic matter, which remains immobile in soil and can be incorporated by plants, and thus enter the food chain [56].

By sampling site, the highest concentrations of Cd for the following sites were LCRU 53.9 ± 5.0, LCAR 52.4 ± 6.5, LDUL 35.9 ± 25.3, and LFRE 29.8 ± 19.8 μg∙g^−1^. Meanwhile, the sites with the lowest concentrations of Cd were S9 2.7 ± 0.4, S6 3.1 ± 1.4, S4 3.4 ± 0.8, and LCOR 3.5 ± 0.9 μg∙g^−1^. In contrast to the results of this research, researchers reported significant difference by climate season and not by sampling site for most of the metals analyzed in the same study area. Cd maximum and minimum values [2] showed significant statistical differences by multiple comparison of means (*p* < 0.05) among the Frente Lagoon–Dulce Lagoon (LFRE-LDUL) and LCAR-LCRU sites; that is, these pairs of sampling sites presented an overlap of means (Figure 2). The rest of the sampling sites, such as S1 to S11, LCOR, LCOL, and LDUL2, did not report significant statistical differences (*p* > 0.05) and there was a value of MSE of 62.662. The average concentrations in the Palizada River in this investigation were higher compared to other investigations carried out in this same area, such as [2], which reported that the Cd maximum concentrations during the rainy season were 2.3 μg∙g^−1^, followed by 2.2 in the north winds season and the dry season with 1.6 μg∙g^−1^ (Table 2). The variations in the concentrations can be attributed to differences in levels of introduction of this element to the aquatic environment, which occur by the anthropogenic route and by dragging pollutants from the source that generates it. 

The Cd for each season did not show significant statistical difference by multiple comparison of means (*p* > 0.05); the MSE value is 326.04; studies reported that this can be associated with a constant contribution of this element to the Palizada River throughout the year [36]. The dry season in the present investigation showed a maximum concentration of 16.7 ± 20.8, followed by the rainy season with 14.8 ± 9.0 and in the north winds with 9.0 ± 13.3 μg·g^−1^. The previous concentrations were higher than those reported in rivers, such as the Palizada with 0.2, Chumpan with 0.266, and Candelaria with 0.2 μg∙g^−1^, the climatic season has a greater influence on the presence and distribution of some heavy metals [36].

The difference in metal concentration can be associated with the type of element; Pb presented a difference between seasons, in contrast to Cd. There were coincidences in sites with maximum values of Pb and Cd for the LCAR and LDUL sites, while the minimum concentrations were for sites S1 and S6. The similarity in the behavior of metals is these sites could be associated with the common sources of these elements and characteristics of the metal. However, researchers have highlighted the difficulty of defining the exact source of some heavy metals, some of these such as Cd, Cu, and Cr, among others, increase the base concentrations considered natural by anthropogenic activities that contribute to these elements in fluvial–lagoon systems such as the Palizada [6]. Likewise, the analysis of sediments in an aquatic ecosystem provides a comprehensive assessment of a site’s contamination [2]. Sediments are main receptors due to the precipitation of most of the pollutants deposited in the water column. The presence of maximum concentrations of both metals is relevant information for its effects on public health, because they are not required for metabolism and are toxic in low concentrations [36].

### 3.2. Geoaccumulation Index (Igeo) in the Palizada Rriver

**Figure 3:**
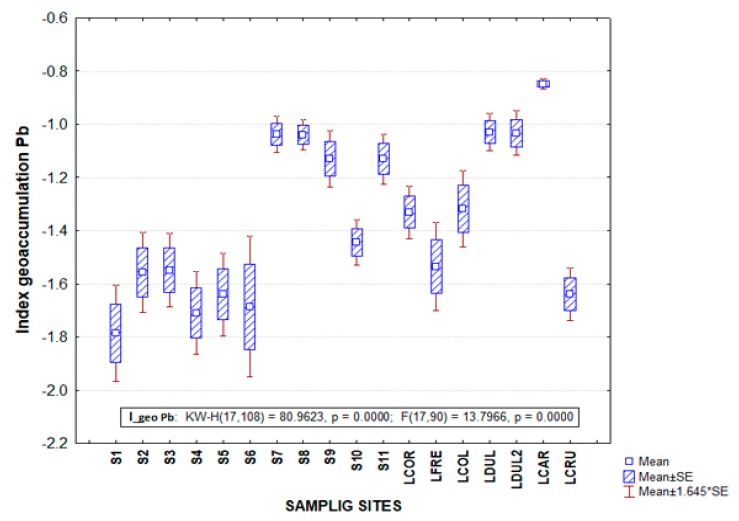
Igeo of heavy metal (Pb) in sediments of the fluvial–lagoon–deltaic system of the Palizada River in Campeche, Mexico.

The LCAR site presented maximum values of Igeo Cd with a value of 7.4, this positioned it in Class 6 with *Igeo* > 5, which is classified as extremely contaminated. In the case of Pb in the same place, it also showed maximum values of both CF of 0.8 and *Igeo* with −0.8 (Table 3), placing it in Class 0, classifying it as practically uncontaminated (Figure 3). The differences in the scale considered for Cd and Pb are associated in addition to the variation of the exclusive reference value for each metal, the sources of pollution in the region due to the anthropogenic contribution from livestock and agricultural activities, as well as the hydrological conformation of Palizada fluvio–lagoon system in numerous internal lagoons influenced by the anthropogenic contribution of pollutants [2]. However, the difficulty of differentiating between anthropogenic and natural contributions of heavy metals in aquatic ecosystems should be highlighted; it is particularly difficult to determine the flow of pollutants when there is an increase in concentrations between seasons [6].

**Figure 4:**
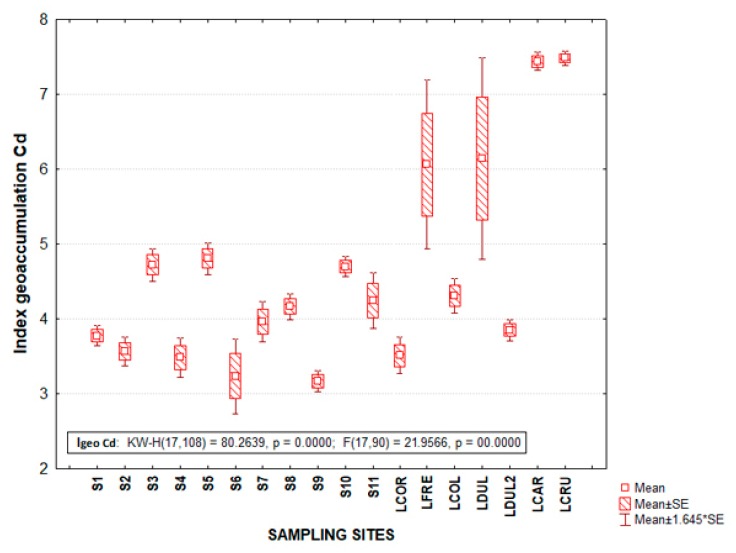
Igeo of heavy metal (Cd) in sediments of the fluvial–lagoon–deltaic system of the Palizada River in Campeche, Mexico.

The Igeo values showed that Cd contributes with the highest enrichment of all the elements analyzed, which showed that there was high contamination in the sediments of the analyzed sites during the two periods of study [57]. The highest levels of Cd may be related to the increase in human and industrial activities in the vicinity of the lagoon in this study; however, Pb concentrations were lower than those reported in other investigations in the case of the Palvio fluvio–lagoon system (Figure 4). An environmental quality estimator such as Igeo has not been used in research previously carried out in the study area [2,36]. The lack of information on the study area makes it difficult for the region and should therefore be compared with research from other regions of the world that may have a totally different heavy metal distribution dynamic. For example, the results of Igeo in the dry season showed different contamination levels of the Ghana River, of which the Cd values stood out as unclogged to highly polluted and the Pb was moderately to extremely contaminating, while the Cr values were considered as moderately contaminating [34]. Differences in metal values indicated a variability of heavy metal enrichment sources. In contrast to the previous study, quantified concentrations of Cd in some of the sites analyzed in their research were included in the class of Igeo 1 (uncontaminated to moderately contaminated) [58,59]. Meanwhile, other sites also analyzed were cataloged within the class of Igeo 3 (moderate to heavily polluted) [58].

The same trend was presented with higher Igeo Cd values with respect to Pb in the study area of this research (Figure 4), which was reported with a minimum value of Pb with −0.08 in the rainy season and in the case of Cd, a maximum value of 5.75 in the dry season [57]. Researchers also reported in the Tsurumi River of Japan Igeo values for Zn, Cu, Cd, Pb, Br placing them in class 0 to 4, which showed a sediment quality that ranges from moderate to heavily contaminated [60]. The differences in sediment quality indexes such as Igeo are indicators of the association between values obtained in the study area with permissible limits, which define the degree of contamination in the analyzed area.

### 3.3. Heavy Metal Pollution Load Index (PLI)

The LCAR site presented maximum CF values of Cd with 262.1 and a PLI with 360.9; this metal contributed the highest value to the PLI. Cd also presented the maximum CF values of all the sampling sites analyzed. The PLI values in the study area were higher than those reported by other investigations, such as those carried out in the Tsurumi River sediments, which reported an average PLI value of 4.88 and minimum and maximum values of 1.24 and 7.65, respectively [60]. Reported levels of PLI confirmed that the quality of water and sediments was classified as contaminated and deteriorating, because they can have a severe impact on the marine and coastal living conditions due to their effects on aquatic organisms [60].

Researchers reported lower PLI values in the Köyceğiz Lagoon System, but on the other hand, they indicated variations between seasons with values of 0.04 to 4.6 during the fall, while in summer, spring, and winter, the minimum values were 0.01, and the maximums of 6.7, 8.4, and 18.6, respectively [61]. These figures also prove that higher levels of PLI in sampling sites can be associated with the discharge of wastewater; this coincides with the differences in values in this research and are related to pollution sources of this same nature [61]. The use of metal contamination indexes can contribute to knowing the health status of an ecosystem, the PLI can provide some understanding of the quality of the environment, and the trends according to time and area also provide valuable information for making decisions about the pollution status of the area [62].

### 3.4. Permissible Limits in Sediments

Evaluating the presence of heavy metals in sediment is a tool that allows us to know the mobilization of these pollutants and their effects on biota, taking into account that sediments are a reservoir of heavy metals that contribute to the degree of bioavailability free for aquatic organisms. The migration of metallic compounds from the abiotic environment to aquatic organisms occurs in sediments, as well as their subsequent introduction and bioaccumulation in trophic chains [48,63]. 

In the case of Mexico, there is no official legislation to regulate concentrations of metals in river sediments [64]. The use of international criteria such as that established [65] helps to deduce the minimum threshold concentrations (ERL) and maximum (ERM) of metals and metalloids that can produce adverse biological responses (Table 4). According to the comparison of the values of ERL, ERM, TRVs, and TEL, Cd concentrations were the only ones that exceeded the established limits of the minimum and maximum thresholds; these results are an indicator of a potential risk for the fluvial–lagoon–deltaic system of the Palizada River by this metal (Table 4). In the Palizada River, Cd values exceed the limits established by the ERL and were higher than international standards [6]. We wish to emphasize the lack of national standards reported that used the ERL value established by the association of this angiosperm plant, with the sediment in grass Thalassia testudinum of prairies of the Términos Lagoon system. In contrast, concentrations of Pb in all the sampling sites of this investigation were lower than the values of ERL and TEL, since the maximum value recorded in the study area was 10.4 ± 0.2 μg∙g^−1^ [64]. The aforementioned value was lower than the ERL of 46.7 μg·g^−1^ [65] and mean of 34.08 mg∙kg^−1^ in sediment of China [66], which indicates that there is no acute risk, yet there coud be a chronic risk due to the bioaccumulation of this element.

The presence of chronic concentrations in the environment can be associated with a risk to public health; prolonged exposure of adults to Pb can cause a deterioration of some functions of the nervous system; plus, it is also listed as a probable carcinogen in humans [46]. In the case of Cd, prolonged exposure to lower levels of Cd in the air, food, and water causes an accumulation of cadmium in the kidneys, is associated with causing kidney disease, and is also classified as a probable carcinogenic in humans [56]. This indicates that its presence in the environment in high concentrations represents a risk to public health and the environment, because plants, fish, and other animals can incorporate Cd and Pb from the environment inside their body [46,56]. 

Other investigations pointed out differences between the threshold values of sampling sites and metals. In sediments from estuaries of southwest India, the minimum values of ERL exceeded these levels in 25 stations for Zn, 29 stations for Cd, 34 stations for Ni, and 41 sites for Pb; these concentrations can cause adverse effects in biological organisms [23]. Pb values in this investigation were found to bewithin international ranges for sampling sites and seasons; in contrast, those of Cd in some of these sampling points surpassed these reference values (Table 4). Consequently, the study of sediments in an aquatic ecosystem allows an integral estimation of pollution, since they are the main receptors of most of the pollutants that are deposited in the water column [2,36].

## 4. Conclusions

The Cd concentrations obtained in the sampling sites of the fluvial–lagoon–deltaic Palizada system presented values that exceeded the ERL and ERM; said reference values according to [65] for heavy metals can cause biological effects in exposed marine organisms. Therefore, the presence of elevated concentrations of Cd in sediment in the zone indicates that they act as integrators of these elements and are a source of transfer to aquatic organisms. The differences in these concentrations and their association with different sources of pollution, the hydrodynamic characteristics of the same system, and the chemical properties of the metals are highlighted. The evaluation of the concentration of heavy metals such as Pb and Cd is a tool for decision making on river–lagoon environments such as the Palizada River, which receive contributions from different origins and can generate sublethal effects on organisms and public health through prolonged exposure to these elements.

On the other hand, Pb presented concentrations lower than the reference values. Its presence means a source of chronic exposure for organisms that inhabit the study area. It also has an effect on the loss and condition of aquatic species, apart from the effect on public health through the trophic chain, and the socioeconomic and environmental importance of this aquatic system.

In addition, Igeo Pb presented values lower than 1, which allows them to be classified as non-contaminated. In contrast, values of Igeo Cd were in the range of moderately contaminated to highly contaminated in the sampling sites analyzed, indicating the existence of contributions with a fundamental androgenic origin with this element. The presence of both metals indicated the incorporation of these elements through diverse sources such as those of an agrochemical type. The deposition of atmospheric and industrial pollutants together contributes to the enrichment of sediments in the analyzed zone. Due to the aforementioned, constant monitoring is required to help identify priority pollution sites to define the magnitude of anthropogenic influence, as well as to establish regulations and policies that reduce the use of compounds that contribute to the incorporation of heavy metals into the region.

## Figures and Tables

**Table 1:** Heavy metals concentration (mean ± standard deviation) in sediment (μg·g^−1^) of the fluvial–lagoon–deltaic system of the Palizada River in Campeche, Mexico.

Site	Pb	Cd
**S1**	5.5 ± 1.0	4.1 ± 0.5
**S2**	6.4 ± 0.9	3.6± 0.6
**S3**	6.4 ± 0.9	8.0 ± 1.6
**S4**	5.7 ± 0.8	3.4 ± 0.8
**S5**	6.0 ± 0.9	8.5 ± 1.6
**S6**	6.0 ± 1.6	3.1 ± 1.4
**S7**	9.1 ± 0.6	4.8± 1.3
**S8**	9.1 ± 0.5	5.4 ± 0.9
**S9**	8.6 ± 0.9	2.7 ± 0.4
**S10**	6.9 ± 0.6	7.8 ± 1.0
**S11**	8.5 ± 0.8	6.0 ± 2.1
**LCOR**	7.4 ± 0.8	3.5 ± 0.9
**LFRE**	6.5 ± 1.1	29.8 ± 19.8
**LCOL**	7.5 ± 1.0	6.0 ± 1.3
**LDUL**	9.2 ± 0.6	35.9 ± 25.3
**LDUL2**	9.1 ± 0.8	4.3 ± 0.6
**LCAR**	10.4 ± 0.2	52.4 ± 6.5
**LCRU**	6.0 ± 0.6	53.9 ± 5.0
**Total**	7.5 ± 1.6	13.5 ± 18.1

**Table 2:** Concentration of heavy metals (mean ± standard deviation) for lead (Pb) and cadmium (Cd) in sediment (μg∙g^−1^) per season of the fluvial–lagoon–deltaic system of the Palizada River in Campeche, Mexico.

Season	Pb	Cd
**R**	7.6 ± 1.5	14.8 ± 19.0
**NW**	6.6 ± 1.7	9.0 ± 13.3
**D**	8.2 ± 1.3	16.7 ± 20.8
**Total**	7.5 ± 1.6	13.5 ± 18.1

**Table 3:** Contamination Factor (*CF*), Pollution Load Index (*PLI*), and Geoaccumulation Index (*Igeo*) of metals in sediments of the fluvial–lagoon–deltaic system of the Palizada River, Campeche, Mexico.

Sampling Site	*CF* Pb	*CF* Cd	*PLI*	*I-geo* Pb	*I-geo* Cd
**Total**	0.6	67.6	71.6	−1.3	4.5
**S1**	0.4	20.7	15.0	−1.7	3.7
**S2**	0.5	18.0	15.6	−1.5	3.5
**S3**	0.5	40.3	35.0	−1.5	4.7
**S4**	0.4	17.2	13.0	−1.7	3.4
**S5**	0.4	42.6	34.6	−1.6	4.8
**S6**	0.4	15.6	11.5	−1.6	3.2
**S7**	0.7	24.1	28.8	−1.0	3.9
**S8**	0.7	27.2	32.9	−1.0	4.1
**S9**	0.6	13.6	15.2	−1.1	3.1
**S10**	0.5	39.1	35.7	−1.4	4.6
**S11**	0.6	30.1	35.1	−1.1	4.2
**LCOR**	0.5	17.6	17.4	−1.3	3.5
**LFRE**	0.5	149.0	138.7	−1.5	6.0
**LCOL**	0.6	30.3	30.9	−1.3	4.3
**LDUL**	0.7	179.8	227.5	−1.0	6.1
**LDUL2**	0.7	21.8	26.7	−1.0	3.8
**LCAR**	0.8	262.1	360.9	−0.8	7.4
**LCRU**	0.4	269.6	216.6	−1.6	7.4

**Table 4:** Permissible limits on the concentration of heavy metals in sediment (mg·kg^−1^).

Permissible Limits	Pb	Cd	Source
Maximum permissible limit (*MPL*)	0.040	0.006	FEPA [67]
Probable effect concentrations (*PEC*)	91.3	3.5	CCME [68]
Interim Sediment Quality Guidelines (*ISQG*)	35	0.6	
Toxicity reference values (*TRVs*)	31	0.60	US EPA [69]
Minimum threshold concentration Effects Range–Low (*ERL*)	46.7	1.2	Long [65]
Maximum threshold concentration Effects Range–Median (*ERM*)	218	9.6	
Threshold effects level (*TEL*)	30.2	0.69	Buchman [70]
Probable effects level (PEL) Threshold effects level (TEL)	112.2 30.2	4.21 0.68	MacDonald et al. [71]
